# Caught in action: how MSCs modulate atherosclerotic plaque

**DOI:** 10.3389/fcell.2024.1379091

**Published:** 2024-03-27

**Authors:** Virginia Egea

**Affiliations:** ^1^ Institute for Cardiovascular Prevention (IPEK), Ludwig-Maximilians-University, Munich, Germany; ^2^ DZHK (German Center for Cardiovascular Research), Partner Site Munich Heart Alliance, Munich, Germany

**Keywords:** MSCs, atherosclerotic plaques, cell-therapy, Trojan horse approach, migration

## Abstract

*Atherosclerosis (AS)* is a medical condition marked by the stiffening and constriction of the arteries. This is caused by the accumulation of plaque, a substance made up of fat, cholesterol, calcium, and other elements present in the blood. Over time, this plaque solidifies and constricts the arteries, restricting the circulation of oxygen-rich blood to the organs and other body parts. The onset and progression of AS involve a continuous inflammatory response, including the infiltration of inflammatory cells, foam cells derived from monocytes/macrophages, and inflammatory cytokines and chemokines. *Mesenchymal stromal cells* (*MSCs*), a type of multipotent stem cells originating from various body tissues, have recently been demonstrated to have a protective and regulatory role in diseases involving inflammation. Consequently, the transplantation of MSCs is being proposed as a novel therapeutic strategy for atherosclerosis treatment. This mini-review intends to provide a summary of the regulatory effects of MSCs at the plaque site to lay the groundwork for therapeutic interventions.

## Introduction

Atherosclerosis is a chronic inflammatory reaction of the blood vessel wall caused by dyslipidemia (Weber and Noels, 2011). Inflammation is important in all stages of atherosclerosis, from plaque formation to rupture (Libby, 2021). When the endothelium is dysfunctional, it disrupts the balance between pro-inflammatory and protective pathways, leading to the accumulation of atherogenic lipoproteins. This triggers the release of chemotactic factors that recruit immune cells and promote the formation of atherosclerotic plaques. Despite the availability of appropriate pharmacological and surgical treatment modalities, AS remains the leading cause of cardiovascular death worldwide (Vaduganathan et al., 2022). Current treatment strategies aim to stabilize plaque, suppress inflammation, and lower serum lipid levels. Interestingly, stem cells exhibit a range of effects, including the ability to regulate lipid levels, suppress inflammation, repair damaged tissues, and support hematopoiesis, offering an innovative approach to the treatment of AS (Nauta and Fibbe, 2007; Li et al., 2017). MSC are present in nearly all tissues and originate from various sources such as bone marrow, adipose tissue, and peripheral blood. These cells possess the ability to differentiate into multiple cell types belonging to the mesodermal and myogenic lineages, a process influenced by environmental stimuli (Pittenger et al., 1999). In addition, MSCs produce a wide array of chemokines, cytokines, and growth factors in response to their surroundings, bestowing upon them immunomodulatory and anti-fibrotic characteristics (Nauta and Fibbe, 2007). In the realm of cell-based therapies MSCs are considered to be exceptional candidates (Lalu et al., 2012; Samsonraj et al., 2017). Their capacity to differentiate into different cell types and *in vitro* expansion are well-established (Salem and Thiemermann, 2010). For therapeutic strategies, it may be feasible to manipulate MSCs *in vitro* and reinfuse them into patients, primarily mitigating risk factors linked to the onset of atherosclerosis, predominantly through a paracrine mechanism. In this scenario, the efficient recruitment of cells to the plaque site is of paramount importance.

## Activation and recruitment of MSC to the atherosclerotic plaques

**FIGURE 1:**
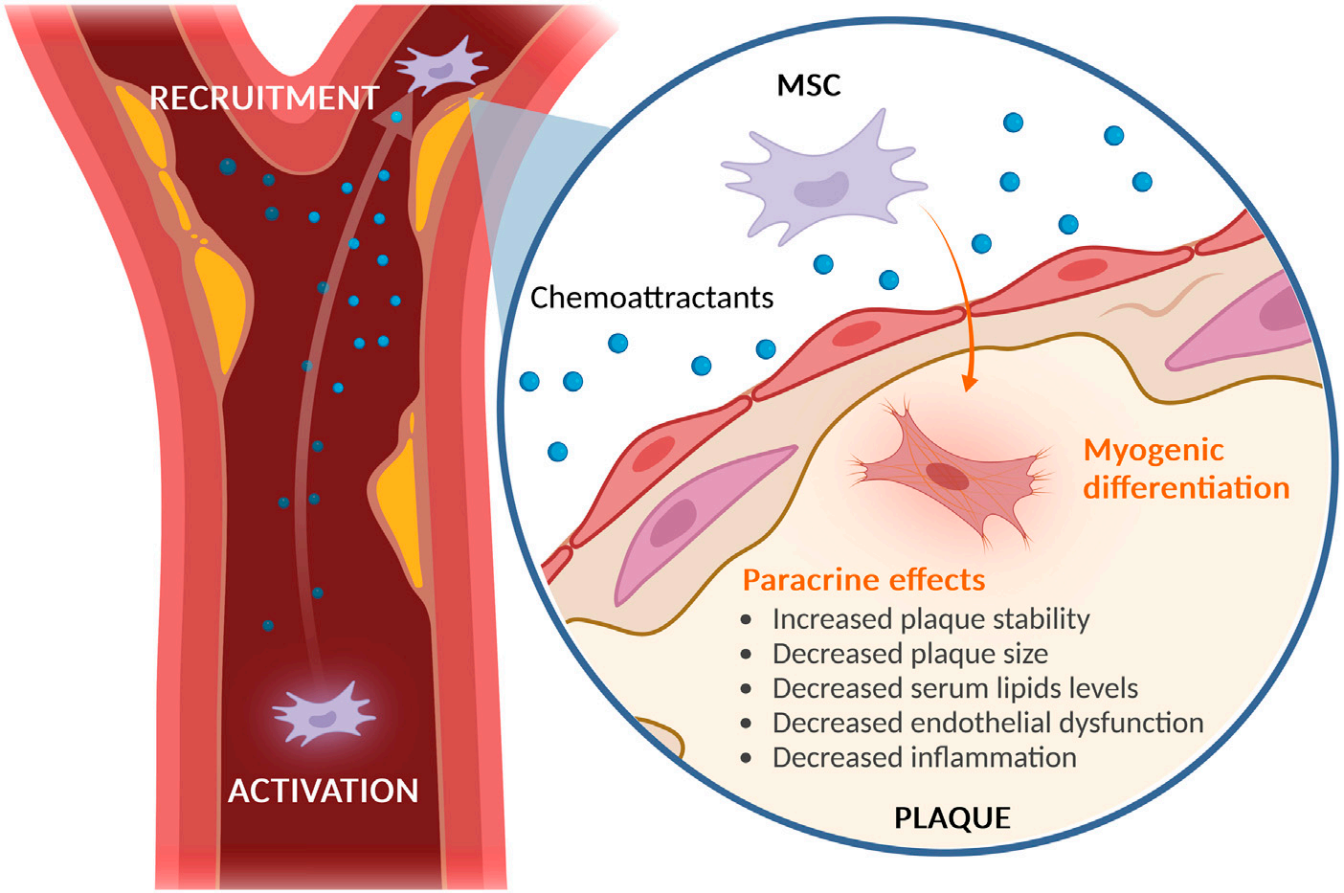
Recruitment of MSCs to Atherosclerotic Plaque. *MSCs* exhibit a natural tendency to migrate to sites of inflammation, including atherosclerotic plaques. Once at these sites, MSCs display a protective role primarily through paracrine signaling, which helps in reducing endothelial dysfunction, hyperlipidemia, and inflammation. This results in an overall increase in plaque stability and decrease in plaque size. Furthermore, studies have shown that MSCs are stimulated by the surrounding atherosclerotic plaque to differentiate into myogenic cells.^11^ Created with BioRender.com.

While the paracrine role of MSCs is increasingly acknowledged, the mechanisms of their migration from the bloodstream to targeted lesions with compromised vascular integrity are not fully understood. Our studies demonstrated that in response to chemotactic signals such as transforming growth factor-beta 1 (TGF-β1), stromal cell-derived factor 1 (SDF-1), interleukin-1 beta (IL-1β) and tumor necrosis factor-alpha (TNF-α), MSCs are able to invade through barriers of extracellular matrix (ECM) facilitated by the secretion of matrix metalloproteinases (MMPs) (Ries et al., 2007). MSCs are capable of migrating through human reconstituted basement membranes, utilizing MMP-2, membrane type 1-MMP (MT1-MMP), and tissue inhibitor of metalloproteinases TIMP-2 for this purpose. TGF-β1 has been identified to increase the levels of MMP-2 and MT1-MMP without affecting TIMP-1 or TIMP-2, indicating its significant role in MSC trafficking through the extracellular matrix (ECM) by inducing these MMPs. Additionally, IL-1β and tumor necrosis factor-alpha TNF-α, which are typically present in wounds and inflamed tissues, have been shown to significantly enhance the expression of MMP-9 and MT1-MMP, thereby promoting MSC invasion. In contrast, SDF-1 has a comparatively minor effect on MSC migration, which may be attributed to a smaller subset of MSCs expressing the C-X-C motif chemokine receptor 4 (CXCR4) receptor or to the MMP-mediated cleavage of SDF-1 (Ries et al., 2007). Nevertheless, blocking the SDF-1/CXCR4 signaling pathway markedly reduces the recruitment of transplanted stem cells to target tissues (Son et al., 2006; Wang et al., 2006). Atherosclerotic plaques, being inflammatory lesions, also generate high levels of cathelicidin antimicrobial peptide LL-37, a small peptide derived from neutrophils which is believed to contribute to disease progression (Zhang et al., 2015). LL-37 has been demonstrated to elevate early growth response factor 1 (*EGR1*) expression and stimulate mitogen-activated protein kinase (MAPK) activation, thereby augmenting MSC functions such as cell proliferation, cell motility, and paracrine activities. These regulatory effects could prove beneficial for tissue regeneration applications, particularly in the context of implantation (Yang et al., 2016). Own recently findings indicate that LL-37, known to be prevalent in the plasma and plaques of atherosclerosis patients, serves as a chemoattractant for MSCs (Egea et al., 2023). Our investigation also identified microRNA (miRNA) let-7f as a pivotal regulator in the LL-37 mediated trafficking of MSCs to inflamed tissues. LL-37 influences cells through formyl peptide receptor 2 (FPR2), a prevalent G protein-coupled receptor that triggers the expression of miRNA let-7f. This, in turn, enhances FPR2 on the cell surface in a positive feedback loop, implying an indirect regulatory function of let-7f by targeting a suppressor of FPR2 expression in these cells (Egea et al., 2023). A similar mechanism of let-7f indirectly boosting CXCR4 expression in MSCs has been previously reported by us. (Egea et al., 2021) Let-7f not only enhances LL-37/FPR2-mediated chemotaxis towards plaques but also upregulates CXCR4, SDF-1α receptor, and induces the expression and release of ECM-degrading MMP-9, thereby improving pericellular proteolysis. This mechanism may facilitate MSCs recruitment in response to tissue injuries and inflammation under physiological conditions (Egea et al., 2021). At the molecular level, let-7f likely targets repressors of cellular susceptibility and chemotactic migration, thereby promoting MSC invasion Figure 1. In recent studies Hu et al. have also shown integrin beta 3 (ITGB3) to improve plaque-recruitment of MSCs into a mouse model of atherosclerosis (Hu et al., 2023).

## Paracrine effects of MSCs in the atherosclerotic plaques

Endothelial dysfunction, the initiator of atherosclerotic plaque formation, involves a positive feedback loop (Marchio et al., 2019). Pathological stimuli such as hypertension cause endothelial damage, leading to the deposition of oxidized LDL (ox-LDL), which triggers an immune response supporting atherogenesis. Nitric oxide (NO), a crucial signaling molecule post-endothelial dysfunction, is produced by endothelial nitric oxide synthase (eNOS) and regulated by Akt-mediated phosphorylation (Fulton, 2016). In atherosclerosis, NO exhibits strong protective effects by inhibiting LDL oxidation, leukocyte adhesion, smooth muscle cell proliferation, and platelet aggregation, while also regulating vascular tone (Marchio et al., 2019).

MSCs have demonstrated the ability to restore endothelial function, thereby stopping atherogenesis (Salvolini et al., 2010). Culture medium from human skin-derived MSCs increased NO production in human aortic endothelial cells, showcasing their paracrine potential (Salvolini et al., 2010). Lin et al. showed that human MSCs prevent ox-LDL-mediated inhibition of eNOS activity in human umbilical vein endothelial cells by phosphorylating and restoring Akt/eNOS activity (Lin et al., 2015).

Furthermore, various studies highlight MSC´s ability to reduce hyperlipidemia, a condition that increases the risk of atherosclerosis, in various animal models (Hong et al., 2019; Libby, 2021). Frodermann et al. reported for instance that using bone marrow-MSCs (BM-MSCs) significantly reduced serum cholesterol levels, particularly very-low-density-lipoproteins (VLDLs), in LDLR−/− mice, 4 weeks post-administration (Frodermann et al., 2015). In a different study, Hong et al. observed that the administration of gingival-MSCs to ApoE−/− mice resulted in a decrease in total cholesterol and LDLs (Hong et al., 2019). They also recorded a reduction in the expression of sterol regulatory element-binding protein 1c (SREBP-1c), a transcription factor involved in fatty acid biosynthesis, and an increase in the expression of peroxisome proliferator-activated receptor-α (PPAR-α), a transcription factor that controls fatty acid β-oxidation. These observations suggest a unique mechanism for MSC-mediated lipid reduction. This theory was further supported by Li et al., who found that the administration of umbilical cord blood-MSCs to leptin-deficient mice resulted in a decrease in lipid levels (Lin et al., 2015). They attributed this to an increase in PPAR-α and a decrease in fatty acid synthase, an enzyme regulated by SREBP-1c, which aligns with Hong et al.'s findings. In summary, there is compelling evidence that the administration of MSCs can reduce serum lipid levels, thereby decreasing lipid accumulation in plaques. However, further research is required to fully comprehend the mechanisms involved and to confirm these results in humans.

Various risk factors such as aging, hypertension, hypercholesterolemia, diabetes, and obesity contribute to the inflammatory onset in AS by promoting the accumulation of ox-LDL, activation of NLRP3 inflammasome, and the recruitment of leukocytes into the plaque (Hoseini et al., 2018). Recent studies have highlighted the anti-inflammatory properties of MSCs, modulating the response of immune cells in the plaque (Lee and Song, 2018).

*Dendritic Cells (DCs) are a type of antigen-presenting cells that significantly contribute to the activation of adaptive immunity* (Gil-Pulido and Zernecke, 2017). *They play a pivotal role in atherogenesis, as they prime and activate T cells.* Research indicates that MSCs can inhibit the differentiation and maturation of DCs, impair antigen uptake, and decrease the expression of costimulatory molecules (CD80, CD86), thereby reducing T-cell activation and proliferation (Reis et al., 2018). This effect is associated with the secretion of extracellular vesicles containing miRNA-21-5p by MSCs (Roufaiel et al., 2016). *T-lymphocytes* represent another cell type influenced by MSCs. Studies have shown that BM-MSCs can reduce DC-induced CD2^+^ T-cell proliferation in a dose-dependent manner, through the secretion of anti-inflammatory cytokines such as TGF-β (Di Nicola et al., 2002). Additionally, cell-to-cell contact has been found to enhance this inhibitory effect. However, the necessity of cell-to-cell contact in reducing T-cell proliferation remains a topic of debate.

*Monocytes and macrophages* are also being influenced by MSCs. Chemotaxis drives monocyte migration from the blood and adventitia into the intima, where they differentiate into macrophages. These mature macrophages can engulf LDLs and become foam cells (Libby, 2021). Several studies have demonstrated that MSCs can reduce macrophage foam cell formation *in vitro* by modulating the expression of scavenger receptors, including CD36, SRA1, and ATP-binding cassette transporter (Wang et al., 2015). Furthermore, MSCs have been shown to reduce the expression of chemokine receptors on inflammatory monocytes and promote phenotype switching to anti-inflammatory macrophages. *In vivo*, the promotion of anti-inflammatory cytokine profiles and suppression of macrophage numbers by MSCs have been used to explain the reduction in plaque size (Li et al., 2015; Zhang et al., 2018).

## Differentiation of MSCs within atherosclerotic plaques

MSC have the ability to differentiate into a variety of cell types, including osteoblasts, chondrocytes, myocytes, and adipocytes (Pittenger et al., 1999). Upon recruitment to the plaque site, these MSCs are subjected to the influences of the new environment, which potentially jeopardizes their stemness and triggers differentiation. Recently, our studies provide evidence that human plaque components elicit differentiation of MSCs into smooth muscle cell-like cells (Egea et al., 2023). The role of MSCs in plaque development is complex and bidirectional. Early in plaque formation, MSCs contribute to inflammation and foam cell formation, while in later stages, they produce extracellular matrix proteins and collagen fibers, which help stabilize the plaques (Bennett et al., 2016). Interestingly, vulnerable plaques prone to rupture are characterized by only few smooth muscle cells in a thin cap (Finn et al., 2010). Thus, myogenic differentiation of MSCs in plaque environment might confer a stabilizing effect in vulnerable plaques in later stages of AS. In fact, transplanted MSCs were shown to stabilize vulnerable plaques in an animal model of atherosclerosis by strengthening the fibrous cap (Wang et al., 2015). Consistently, we found that human plaque lysates upregulated endogenous levels of miR-335 in MSCs, a miRNA shown to promote overall plaque stability (Egea et al., 2023).

## Conclusion and future perspective

Numerous research efforts have highlighted the propensity of MSCs to migrate towards atheromatous tissues, contributing to atheroprotection through the secretion of paracrine factors and their maturation into cells that stabilize plaques. The differentiation potential, paracrine effects, exosomal release, and direct-contact modulatory functions of MSCs have been the focus of extensive investigation. Each of these mechanisms plays a role in the holistic process of MSC therapy in AS. Nevertheless, the protective mechanisms of MSCs warrant further exploration. Understanding the function of MSCs in varying stages of atherosclerosis, the disparities among MSC sources, and the efficiency of MSC recruitment to the plaque are all crucial areas for further study to enhance the safety, efficacy, and outcomes of MSC-based therapy.
